# Prevalence, Antimicrobial Resistance Profile, and Genetic Characteristics of Methicillin-Resistant *Staphylococcus aureus* Isolated from Retail Raw Fish in South Korea

**DOI:** 10.3390/microorganisms13061415

**Published:** 2025-06-17

**Authors:** Haiseong Kang, Jonghoon Lee, Ji Min Han, Yong hoon Kim, Insun Joo, Hyochin Kim

**Affiliations:** Food Microbiology Division, Food Safety Evaluation Department, National Institute of Food and Drug Safety Evaluation, Cheongju 28159, Republic of Korea; whdgns46@korea.kr (J.L.); jimin9746@korea.kr (J.M.H.); washout71@korea.kr (Y.h.K.); jis901@korea.kr (I.J.)

**Keywords:** methicillin-resistance *Staphylococcus aureus*, antibiotic resistance, whole-genome sequencing, raw fish

## Abstract

Given the limited genetic characterization of methicillin-resistant *Staphylococcus aureus* (MRSA) in South Korea, we performed whole-genome sequencing (WGS) on eight MRSA strains isolated from raw fish products, including 327 sliced raw flatfish, 111 flatfish stew products, 85 sliced raw rockfish, and 11 rockfish stew products. Phylogenetic analysis revealed two distinct clusters—comprising five ST72-t324-SCC*mec*IVc strains and two novel sequence types—as well as a singleton strain (ST630-t4549-SCC*mec*V). A total of five antimicrobial resistance genes (ARGs), four plasmid replicon genes, three mobile genetic element genes, and seventy-three virulence factor genes were identified, with distinct genetic profiles observed between the clusters. Notably, several MRSA strains were isolated from samples obtained at the same retail market, indicating potential local clustering. Four ST72-t324-SCC*mec*IVc strains, collected from the same market, shared identical profiles in terms of four ARGs, two plasmid replicons, two mobile genetic elements, and several virulence factor genes. These findings provide valuable insights into the genomic characteristics of MRSA in raw fish products and highlight the need for ongoing surveillance and monitoring efforts in South Korea.

## 1. Introduction

Korea’s per capita aquatic food consumption (68.4 kg per capita per year) is significantly higher than the global average (20.6 kg per capita per year) [1,2]. In Korea, flatfish and rockfish are among the most commonly consumed raw fish, with 48% of flatfish being farmed [2]. Antibiotics are commonly used in fish farming for both therapeutic and prophylactic purposes. To regulate antibiotic residues in farmed fish, the Korean government enforces mandatory withdrawal periods before the products can be marketed [3]. However, fish transported to retail markets can become contaminated with foodborne pathogens if they are raised in polluted waters or if hygiene protocols are not properly followed during transport, storage, and handling [4]. Therefore, monitoring seafood for the presence of foodborne pathogens is essential.

*Staphylococcus aureus* (*S. aureus*) is a common bacterium found on human skin and mucosal surfaces and can occasionally cause infections [5]. However, methicillin-resistant *S. aureus* (MRSA) is a serious pathogen, resistant to multiple antimicrobials, associated with difficult-to-treat infections, and easily transmissible [6]. Although *S. aureus* is not part of the normal fish microbiota [7], it exhibits high tolerance to salt stress and can survive in aquatic environments and salted foods [8]. In particular, aquaria contaminated with MRSA may act as reservoirs, contributing to community transmission and facilitating the spread of antimicrobial resistance genes through horizontal gene transfer [9].

MRSA is traditionally classified by *spa* typing, staphylococcal cassette chromosome *mec (*SCC*mec*) typing, and multilocus sequence typing (MLST). However, whole-genome sequencing (WGS) provides high-resolution analysis at the gene level, facilitating the identification of antibiotic resistance genes, plasmid replicons, and virulence factors [10,11]. Therefore, WGS offers valuable data for the tracking and characterization of foodborne zoonotic bacterial pathogens.

Despite Korea’s high level of aquatic food consumption, genetic characterization data for MRSA in seafood remain limited. Given this gap, this study aimed to isolate MRSA strains from South Korean aquatic food and characterize their genetic features using WGS. We isolated eight MRSA strains and assessed their susceptibility to 15 antimicrobial subclasses. In addition, we performed phylogenetic analyses based on MLST, SCC*mec* typing, and *spa* typing. Genes related to antimicrobial resistance (AMR), plasmid replicons, mobile genetic elements (MGEs), and virulence factors (VFs) were also analyzed. Our findings provide valuable insights into the genetic characteristics of MRSA in aquatic food and contribute to efforts in public health surveillance and food safety.

## 2. Materials and Methods

### 2.1. Sample Collection

Between February 2022 and November 2023, a total of 534 raw fish samples were collected from retail markets in Korea. These included 327 sliced raw flatfish, 111 flatfish stew samples, 85 sliced raw rockfish, and 11 rockfish stew samples. The stew samples comprised fish heads, guts, eggs, and bones that remained after slicing. Samples were obtained from 24 cities across five regions and were immediately refrigerated and transported to the laboratory. All fish were farmed in aquaculture facilities.

### 2.2. Isolation and Identification of Staphylococcus aureus

*Staphylococcus aureus* was isolated according to the method outlined in the MFDS Food Code [12]. Briefly, 25 g of each sample was homogenized with 225 mL of tryptic soy broth (Oxoid, Basingstoke, UK) containing 10% NaCl, incubated at 37 °C for 48 h. The cultures were then streaked onto Baird–Parker agar (MB cell) with egg yolk tellurite and incubated at 37 °C for an additional 48 h. Presumptive *S. aureus* colonies were subcultured on Tryptone Soya Agar (Oxoid) and incubated at 37 °C for 24 h. Final identification was performed using matrix-assisted laser desorption ionization-time of flight (MALDI-TOF) mass spectrometry with the VITEK MS PRIME system (bioMérieux, Marcy-l’Étoile, France). One isolate per sample was selected, and confirmed *S. aureus* strains were stored at −80 °C in tryptic soy broth with 10% glycerol.

### 2.3. Antimicrobial Susceptibility

All *S. aureus* isolates were subjected to a minimum inhibitory concentration (MIC) assay. The test was performed using the EUST2 panel (TREK Diagnostic Systems, Cleveland, OH, USA), following the manufacturer’s instructions. *S. aureus* ATCC 29213 was used as a quality control strain. Each panel contained 17 antimicrobial agents from 15 different subclasses. MIC results were interpreted based on the guidelines provided by the Clinical and Laboratory Standards Institute (CLSI) [13]. As CLSI does not provide breakpoints for mupirocin and fusidic acid, the European Committee on Antimicrobial Susceptibility Testing (EUCAST) criteria were applied for these agents [14].

### 2.4. Identification of Methicillin Resistant Staphylococcus aureus

Cefoxitin-resistant isolates were screened for the presence of the *mecA* gene by PCR. Genomic DNA was extracted using the MagListo 5M Genomic DNA Extraction Kit (Bioneer, Daejeon, Republic of Korea), according to the manufacturer’s protocol. The primer sequences used were as follows: *mA1* (TGCTATCCACCCTCAAACAGG) and *mA2* (AACGTTGTAACCACCCCAAGA) [15]. The PCR conditions were as follows: initial denaturation at 95 °C for 5 min, followed by 28 cycles of 97 °C for 10 s, 56 °C for 20 s, and 72 °C for 60 s, and a final extension at 72 °C for 7 min. PCR products were visualized by 2.0% agarose gel electrophoresis. Only *mecA*-positive isolates were subjected to whole-genome sequencing.

### 2.5. Whole-Genome Sequencing and Sequence Analysis

Eight *mecA*-positive isolates were selected for whole-genome sequencing. Genomic DNA was extracted using the MagListo 5M Genomic DNA Extraction Kit (Bioneer, Daejeon, Republic of Korea). Libraries were prepared using the Illumina DNA Prep Kit and sequenced on the Illumina MiSeq platform (Illumina Inc., San Diego, CA, USA) with paired-end reads of approximately 600 bp. Trimming was performed using Trimmomatic (version 0.38), and de novo assembly was conducted using SPAdes (version 3.13.0) and CLC Genomics Workbench (version 12). Contigs shorter than 1000 bp and with a sequencing depth below 5× were excluded from downstream analysis.

### 2.6. Nucleotide Sequence Accession Numbers

Raw sequencing data have been deposited in GenBank under BioProject PRJNA1196158, with the following BioSample accession numbers: SAMN45375310 (2022_STA_378), SAMN45375311 (2022_STA_477), SAMN45375312 (2022_STA_1566), SAMN45375313 (2022_STA_1567), SAMN45375314 (2022_STA_1568), SAMN45375315 (2022_STA_1569), SAMN45375316 (2023_STA_594), and SAMN45375317 (2023_STA_595).

### 2.7. Phylogenetic Analysis Using Whole-Genome Sequencing

Phylogenetic classification was conducted using *spa* typing, SCC*mec* typing, multilocus sequence typing (MLST), core genome MLST (cgMLST), and single nucleotide polymorphism (SNP) phylogeny. *Spa* typing was performed using *spa*Typer (version 1.0), and SCC*mec* typing was performed with SCC*mec*Finder (version 1.2). MLST, cgMLST, and clonal complexe were obtained with those determined and compared in PubMLST online database [16]. SNP analysis was performed using CSI Phylogeny (version 1.4), and a minimum-spanning tree was constructed using GrapeTree (version 1.5.0). Eight MRSA isolates and the reference genome *S. aureus* US500 (accession number CP000255) were used.

### 2.8. In Silico Characterization of Whole-Genome Sequences

WGS data were used to characterize plasmid replicons, antimicrobial resistance genes, point mutation, mobile genetic elements, and virulence factors. PlasmidFinder (version 2.1) was used with thresholds of ≥95% identity and ≥60% coverage. Antibiotic resistance genes and point mutation were identified using ResFinder (version 4.7.2) and PointFinder (database 4.1.1) under thresholds of ≥90% identity and ≥60% coverage, respectively. Mobile genetic elements were predicted using MobileElementFinder (software version 1.0.3, database version 1.0.2). Virulence genes were identified using the Virulence Factor Database (VFDB; http://www.mgc.ac.cn/cgi-bin/VFs/v5/main.cgi?func=VFanalyzer, accessed on 5 December 2024) with thresholds of ≥90% identity and ≥50% coverage.

## 3. Results

### 3.1. Prevalence of Staphylococcus aureus and Methicillin-Resistant Staphylococcus aureus in Raw Fish Samples

The prevalence rates of *S. aureus*, cefoxitin-resistant *S. aureus*, and *mecA*-positive MRSA are summarized in Table 1. *S. aureus* was detected in 11.0% (*n* = 36) of sliced raw flatfish (*n* = 327), 7.2% (*n* = 8) of raw flatfish stew (*n* = 111), 9.4% (*n* = 8) of sliced raw rockfish (*n* = 85), and 18.2% (*n* = 2) of raw rockfish stew (*n* = 11). Cefoxitin-resistant *S. aureus* was found in 1.5% (*n* = 5), 1.8% (*n* = 2), 1.2% (*n* = 1), and 18.2% (*n* = 2) of the same sample types, respectively. Cefoxitin-resistant *S. aureus* isolates were subjected to *mecA* PCR assay. *MecA*-positive MRSA was confirmed in 0.9% (*n* = 3) of sliced raw flatfish, 1.8% (*n* = 2) of raw flatfish stew, 1.2% (*n* = 1) of sliced raw rockfish, and 18.2% (*n* = 2) of raw rockfish stew.

### 3.2. Antimicrobial Susceptibility and Confirmation of MRSA

A total of 54 *S. aureus* strains were subjected to antimicrobial susceptibility testing against 17 agents belonging to 15 antimicrobial subclasses (Table 2). Among these, the highest resistance was observed against penicillin (72.2%), followed by fusidic acid (44.4%), ciprofloxacin (20.4%), cefoxitin (18.5%), and tetracycline (11.1%). All isolates were susceptible to linezolid, mupirocin, rifampin, streptomycin, tiamulin, and vancomycin. Cefoxitin-resistant strains (*n* = 10) were further subjected to PCR analysis for the *mecA* gene. Among them, eight strains were confirmed to be *mecA*-positive MRSA.

### 3.3. Antimicrobial Susceptibility Profiles of MRSA

All MRSA isolates were resistant to both cefoxitin and penicillin (Table 3). In addition, resistance was observed in two isolates against kanamycin, one against ciprofloxacin, and one against fusidic acid. Four strains exhibited a multidrug-resistant (MDR) phenotype, defined as resistance to three or more antimicrobial subclasses. The remaining four strains were resistant only to cefoxitin and penicillin. 

### 3.4. Phylogenetic Analysis

**Figure 1:**
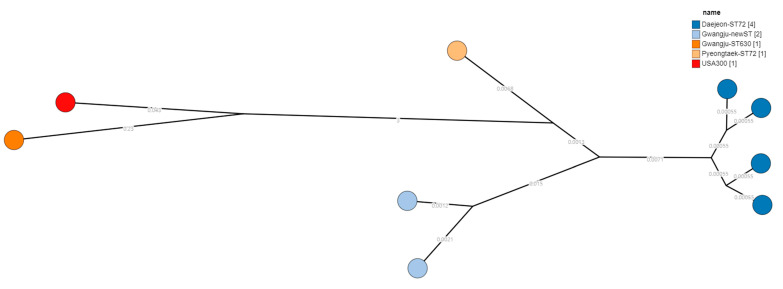
Single nucleotide polymorphism-based minimum-spanning tree.

**Figure 2:**
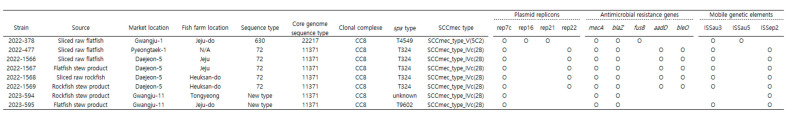
Identified sequence type, core genome sequence type, clonal complexes, *spa* type, SCC*mec* type, plasmid, antimicrobial resistance, and mobile genetic element gene profile of MRSA.

As a result of SNP analysis, two clusters and two singletons were classified (Figure 1). Four Daejeon-ST72 strains and two Gwangju-newST were divided cluster, respectively. Gwangju-ST630 and Pyeongtaek-ST72 were divided singleton, respectively. Daejeon-ST72 cluster was identified ST72-cgST11371-CC8-T324-SCC*mec*IVc (Figure 2). Gwangju-newST cluster was identified newST-cgST11371-CC8-SCC*mec*IVc. Gwangju-ST630 singleton was identified ST630-cgST22217-CC8-t4549-SCC*mec*V. Pyeongtaek-ST72 singleton was identified ST72-cgST11371-CC8-t324-SCC*mec*IVc.

### 3.5. Detection of Antimicrobial Resistance Genes and Plasmid Replicons

Five antimicrobial resistance genes (*mecA*, *blaZ*, *fusB*, *aadD*, and *bleO*), four plasmid replicons (rep7c, rep16, rep21, and rep22), and three mobile genetic elements (ISSau3, ISSau5, and ISSep2) were identified. Daejeon-ST72 cluster and Pyeongtaek-ST72 singleton were determined *mecA*, *blaZ*, *aadD*, and *bleO*, along with rep7c, rep22, ISSau3, and ISSep2. Gwangju-newST cluster was determined *mecA* and *blaZ*, one plasmid replicon (rep7c), and two MGEs (ISSau3 and ISSep2). Gwangju-ST630 singleton was determined *mecA*, *blaZ*, and *fusB*, and three plasmid replicons (rep7c, rep16 and rep21) and two MGEs (ISSau3 and ISSau5).

### 3.6. Detection of Point Mutation AMD Virulence Factors

The Gwangju-ST630 singleton was detected eight point mutations (*dfrB, grlA, grlB, gyrA, ileS, pbp2, pbp4*, and *rpoB*), and the other seven strains detected 11 point mutations (*23S, dfrB, fusA, grlA, grlB, gyrA, ileS, pbp2, pbp4, rpoB*, and *pbp4-*promoter). Comprehensive in silico analysis of virulence factors revealed the presence of genes belonging to five functional classes, comprising 73 virulence-related genes in total. Among these, 38 genes were consistently detected across all isolates (Table 4), while 35 genes exhibited strain-specific variation (Table 5). Toxin-associated genes were the most frequently detected class. The Gwangju-ST630 singleton strain carried 48 virulence genes, Daejeon-ST72 cluster and Pyeongtaek-ST72 singleton carried 61 to 64 virulence genes, and Gwangju-newST cluster carried 58 and 59 virulence genes, respectively.

## 4. Discussion

In this study, *Staphylococcus aureus*, including methicillin-resistant *S. aureus* (MRSA), was isolated from 54 of 534 raw fish samples (327 sliced raw flatfish, 111 flatfish stew products, 85 sliced raw rockfish, and 11 rockfish stew products) collected from 138 retail stores across 24 cities in South Korea between 2022 and 2023. As *S. aureus* is not a component of the normal fish microbiota [8], its presence in raw fish suggests exogenous contamination. Although fish tank environments exhibit high salinity and low temperatures similar to seawater, the prevalence of *S. aureus* and MRSA was significant in these products. Compared to retail meat products, the prevalence of *S. aureus* in raw fish was lower; however, MRSA prevalence was notably higher [17]. Aquatic animals are typically transported and maintained alive in water tanks from aquaculture farms to markets. Antibiotics are frequently used in these systems for disease prevention and growth promotion. However, the extent of their use remains unclear, and residues may persist in aquatic environments. Prolonged antimicrobial exposure can decrease microbial diversity and apply selective pressure, favoring the emergence of resistant bacteria [18]. Therefore, the higher prevalence of MRSA in aquatic animal products compared to terrestrial ones may reflect sustained antibiotic stress in aquatic systems.

*S. aureus* is capable of thriving in aquatic environments due to its tolerance to high salt concentrations (up to 10% NaCl) and wide growth temperature range (7–48 °C) [9]. If contamination occurs in fish tanks, *S. aureus* can proliferate and potentially infect fish. Supporting this, WGS analysis of MRSA-positive samples from specific markets revealed clustering patterns suggestive of localized environmental contamination. Of the eight MRSA strains identified, five were classified as ST72-t324-SCC*mec*IVc, with four of these originating from Daejeon-5 Mart. Notably, these four strains were isolated from different fish types and products; however, they displayed high genetic similarity, implying a common contamination source, likely the fish tank environment or associated handling surfaces. A similar pattern was observed for a newly identified sequence type (New ST2), isolated from a stew product sample from Gwangju-11 Mart. The detection of MRSA only in the processed product suggests contamination may have occurred during preparation using by-products or utensils. These findings highlight potential hygiene issues within retail environments, including the tanks, processing equipment, and worker hygiene. If MRSA contamination is widespread within a mart, the location may serve as a persistent reservoir for community-associated MRSA, increasing the risk of public exposure and infection until hygiene conditions are improved.

Genomic analysis revealed the presence of *mecA*, *blaZ*, and rep7c in all MRSA isolates, with rep7c potentially serving as a key plasmid vector for *mecA*. The ST72-t324-SCC*mec*IVc group additionally carried the rep22 plasmid and resistance genes *aadD* and *bleO*, suggesting that rep22 may be responsible for their mobilization. ST72 is a well-recognized clinical MRSA clone in South Korea, and *spa* type t324 is one of its major variants [19]. The ST72-t324 clone has also been detected in retail meat and clinical and terrestrial animal sources, indicating its widespread presence and possible predominance in Korea. One ST630-t4549-SCC*mec*V isolate was unique in exhibiting fusidic acid resistance at both phenotypic and genotypic levels. Fusidic acid is commonly used as a topical treatment for *S. aureus* skin infections in humans [20]. Its resistance is more prevalent in patients with dermatological conditions such as atopic dermatitis [21]. While wild aquatic organisms tend to be susceptible to fusidic acid, resistance has been reported in farmed fish [22]. These findings raise the possibility that fusidic acid-resistant *S. aureus* in aquaculture may be of human origin.

This study has several limitations. First, only raw fish products were sampled; environmental samples such as tank water, processing surfaces, and worker hands were not included, limiting the ability to pinpoint sources of contamination. Second, methicillin-susceptible *S. aureus* (MSSA) isolates were excluded from WGS analysis. The inclusion of MSSA would have provided a more comprehensive understanding of *S. aureus* population dynamics. Third, comparative analysis was constrained due to a lack of WGS data on MRSA from fish products in South Korea. Nevertheless, this study represents the first WGS-based analysis of MRSA from retail raw fish in South Korea. It provides valuable insights into the genetic characteristics of MRSA, including MLST, cgMLST, SNP, clonal complexe, *spa* type, SCC*mec* type, point mutation, antibiotic resistance genes, plasmid content, mobile elements, and virulence factors.

In conclusion, our findings suggest that community retail markets handling raw fish may act as reservoirs for antibiotic-resistant bacteria, including MRSA. Given *S. aureus*’s tolerance to salinity and temperature fluctuations, contamination of aquatic tanks poses a significant risk. The consumption of raw fish contaminated with MRSA could lead to serious foodborne illnesses that are difficult to treat. Thus, improved hygiene practices and strict monitoring of raw fish handling are essential to reduce the risk of MRSA transmission within the community.

## Figures and Tables

**Table 1:** Sample tested in this study.

Sample Type	Prevalence of Isolates (%; No. of Isolate-Positive Samples/No. of Tested Samples)		
	*S. aureus*	Cefoxitin Resistance *S. aureus*	*MecA*-Positive MRSA
Sliced raw flatfish	11.0 (36/327)	1.5 (5/327)	0.9 (3/327)
Flatfish stew product	7.2 (8/111)	1.8 (2/111)	1.8 (2/111)
Sliced raw rockfish	9.4 (8/85)	1.2 (1/85)	1.2 (1/85)
Rockfish stew product	18.2 (2/11)	18.2 (2/11)	18.2 (2/11)

**Table 2:** Antibiotic resistance profiles of *S. aureus* isolated from raw fish samples.

Antimicrobial Agent	% (Number of Resistant Strains)				
	Sliced Raw Flatfish (*n* = 36)	Flatfish Stew Product (*n* = 8)	Sliced Raw Rockfish (*n* = 8)	Rockfish Stew Product (*n* = 2)	Total
Cefoxitin	14.3 (5)	25.0 (2)	11.1 (1)	100 (2)	18.5 (10)
Chloramphenicol	2.9 (1)	25.0 (2)	0.0 (0)	0.0 (0)	5.6 (3)
Ciprofloxacin	25.7 (9)	12.5 (1)	11.1 (1)	0.0 (0)	20.4 (11)
Clindamycin	2.9 (1)	0.0 (0)	0.0 (0)	0.0 (0)	1.9 (1)
Erythromycin	20.0 (7)	12.5 (1)	0.0 (0)	0.0 (0)	14.8 (8)
Fusidate	48.6 (17)	37.5 (3)	44.4 (4)	0.0 (0)	44.4 (24)
Gentamicin	2.9 (1)	0.0 (0)	0.0 (0)	0.0 (0)	1.9 (1)
Kanamycin	8.6 (3)	0.0 (0)	11.1 (1)	0.0 (0)	7.4 (4)
Linezolid	0.0 (0)	0.0 (0)	0.0 (0)	0.0 (0)	0.0 (0)
Mupirocin	0.0 (0)	0.0 (0)	0.0 (0)	0.0 (0)	0.0 (0)
Penicillin	60.0 (21)	100 (8)	88.9 (8)	100 (2)	72.2 (39)
Quinupristin/Dalfopristin	2.9 (1)	0.0 (0)	0.0 (0)	0.0 (0)	1.9 (1)
Rifampin	0.0 (0)	0.0 (0)	0.0 (0)	0.0 (0)	0.0 (0)
Sulfamethoxazole	5.7 (2)	0.0 (0)	0.0 (0)	0.0 (0)	3.7 (2)
Tetracycline	14.3 (5)	12.5 (1)	0.0 (0)	0.0 (0)	11.1 (6)
Trimethoprim	8.6 (3)	12.5 (1)	0.0 (0)	0.0 (0)	7.4 (4)
Vancomycin	0.0 (0)	0.0 (0)	0.0 (0)	0.0 (0)	0.0 (0)

**Table 3:** Antimicrobial resistance profiles of MRSA.

			Antimicrobial Resistance																
Year	Strain	Source	Cefoxitin	Chloramphenicol	Ciprofloxacin	Clindamycin	Erythromycin	Fusidate	Gentamicin	Kanamycin	Linezolid	Mupirocin	Penicillin	Quinupristin/Dalfopristin	Rifampin	Sulfamethoxazole	Tetracycline	Trimethoprim	Vancomycin
2022	378	Sliced raw flatfish	O					O					O						
2022	477	Sliced raw flatfish	O		O								O						
2022	1566	Sliced raw flatfish	O							O			O						
2022	1567	Flatfish stew product	O										O						
2022	1568	Sliced raw rockfish	O							O			O						
2022	1569	rockfish stew product	O										O						
2023	594	rockfish stew product	O										O						
2023	595	Flatfish stew product	O										O						

**Table 4:** Identified common virulence factor genes of MRSA.

VF Class	Virulence Factor	Related Gene
Adherence	Elastin binding protein	*ebp*
	Fibrinogen binding protein	*efb*
	Fibronectin binding proteins	*fnbA*, *fnbB*
	Intercellular adhesin	*icaA*, *icaB*, *icaC*, *icaR*
	Staphylococcal protein A	*spa*
Enzyme	Cysteine protease	*sspB*, *sspC*
	Hyaluronate lyase	*hysA*
	Lipase	*Geh*, *lip*
	Serine V8 protease	*sspA*
	Staphylocoagulase	*coa*
	Thermonuclease	*nuc*
Immune evasion	Sbi	*sbi*
Secretion system	Type VII secretion system	*esaA*, *esaD*, *esaE*, *esaG*, *essA*, *essB*, *essC*, *esxA*, *esxB*, *esxC*, *esxD*
Toxin	Alpha hemolysin	*hly/hla*
	Delta hemolysin	*hld*
	Exotoxin	*set18*, *set30*, *set31*, *set37*
	Gamma hemolysin	*hlgA*, *hlgB*, *hlgC*

**Table 5:** Identified virulence factor genes of MRSA except common virulence factor genes.

Virulence Factor Class	Virulence Factors	Related Genes	2022_378	2022_477	2022_1566	2022_1567	2022_1568	2022_1569	2023_594	2023_595
Adherence	Autolysin	*atl*	O	O	O	O	O	O		O
	Cell wall associated fibronectin binding protein	*ebh*	O							
	Clumping factor A	*clfA*	O							
	Intercellular adhesin	*icaD*			O	O	O			
	Ser-Asp rich fibrinogen-binding proteins	*sdrC*	O		O	O	O	O		
		*sdrD*			O	O	O	O		
		*sdrE*			O	O	O	O		O
Enzyme	Serine protease	*splA*		O	O	O	O	O	O	O
		*splB*		O	O	O	O	O	O	O
		*splC*		O	O	O	O	O	O	O
		*splD*		O	O	O	O	O	O	O
	Staphylokinase	*sak*		O	O	O	O	O	O	O
Immune evasion	AdsA	*adsA*		O	O	O	O	O	O	O
	CHIPS	*chp*		O	O	O	O	O	O	O
	SCIN	*scn*		O	O	O	O	O	O	O
Secretion system	Type VII secretion system	*esaB*		O	O	O	O	O		O
Toxin	Enterotoxin G	*seg*		O	O	O	O	O	O	O
	Enterotoxin Yent2	*yent2*		O	O	O	O	O	O	O
	Enterotoxin-like K	*selk*		O	O	O	O	O	O	O
	Enterotoxin-like M	*selm*		O	O	O	O	O	O	O
	Enterotoxin-like N	*seln*		O	O	O	O	O	O	O
	Enterotoxin-like O	*selo*		O	O	O	O	O	O	O
	Exotoxin	*set10*								O
		*set15*		O	O	O	O	O	O	O
		*set21*	O							
		*set22*		O	O	O	O	O	O	O
		*set24*		O	O	O	O	O	O	O
		*set25*		O	O	O	O	O	O	O
		*set33*		O						
		*set34*	O	O	O	O	O	O	O	
		*set36*	O							
		*set38*	O							
		*set39*	O							
		*set40*	O							
	Leukotoxin D	*lukD*		O	O	O	O	O	O	O

## Data Availability

All WGS data on the eight isolates are available under NCBI BioProject PRJNA1196158.
